# Cyclo-Graphyne: A Highly Porous and Semimetallic 2D Carbon Allotrope with Dirac Cones

**DOI:** 10.1021/acsomega.5c10456

**Published:** 2026-01-21

**Authors:** Jhionathan de Lima, Cristiano F. Woellner

**Affiliations:** † Department of Physics, 28122 Federal University of Parana, UFPR, Curitiba, PR 81531-980, Brazil; ‡ Interdisciplinary Center for Science, Technology, and Innovation (CICTI), 28122 Federal University of Parana, UFPR, Curitiba, PR 81530-000, Brazil

## Abstract

We present a comprehensive characterization of Cyclo-graphyne (CGY), an emerging 2D carbon allotrope with a porous structure of sp- and sp^2^-hybridized carbon atoms. Using density functional theory, we systematically investigate its structural, energetic, dynamical, electronic, mechanical, optical, and vibrational properties. The calculated cohesive and formation energies are both comparable to those of other synthesized graphynes, confirming its energetic viability. Phonon dispersion calculations confirm its dynamical stability, while ab initio molecular dynamics simulations indicate thermal stability up to at least 1000 K. Electronic structure calculations reveal that CGY is a semimetal featuring two Dirac cones in its electronic structure. Mechanically, this material is highly compliant and isotropic, exhibiting a Young’s modulus an order of magnitude lower than that of graphene. The optical spectrum reveals strong ultraviolet absorption and infrared reflectivity with an isotropic response, while the vibrational spectra show distinct Raman peaks and rich infrared activity. These properties position CGY as a promising candidate for future applications in areas such as gas capture and separation, flexible nanoelectronics, and optoelectronics.

## Graphical abstract

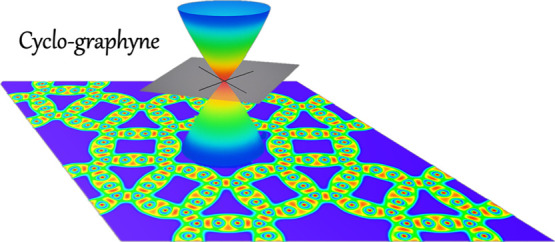

## Introduction

Carbon is a remarkably versatile element due to its ability to adopt different hybridizations, including sp, sp^2^, and sp^3^ configurations. This flexibility enables the formation of a wide variety of carbon allotropes with diverse covalent bonding schemes. Diamond and graphite are the most common natural allotropes of carbon, with hybridization states of sp^3^ and sp^2^, respectively. Beyond these natural allotropes, a wide range of two-dimensional (2D) synthetic carbon nanomaterials has been theoretically predicted and, in some cases, experimentally realized, including graphene,^1^ the biphenylene network,^2^ graphenylene,^3^ γ-graphyne,^4–8^ γ-graphdiyne,^9^ and the fullerene network.^10^

Among the emerging carbon allotropes, graphynes (GYs) represent a particularly intriguing class of 2D nanomaterials, as they integrate both sp- and sp^2^-hybridized carbon atoms within their extended frameworks.^11^ The presence of acetylenic groups reduces areal density and introduces tunable porosity, leading to distinct physical properties.^12^ Several GY allotropes exhibit unconventional electronic band structures characterized by Dirac cones near the Fermi level, including α-,^13,14^ β-,^13,14^ δ-,^15^ R-,^16^ 6,6,12-,^17,18^ 14,14,14-,^18^ 14,14,18-,^18^ 14,12,2-,^19^ 14,12,4-,^19^ cp-GY,^20^ and α-graphdiyne (GDY).^21^ For example, the honeycomb α-GY exhibits graphene-like dispersion with Dirac cones located at the *K* and *K*′ points,^13,14,17,18^ although it is energetically less favorable than other variants. In contrast, β-GY hosts Dirac cones along the Γ–*M* path,^13,17,18^ while 6,6,12-GY presents two nonequivalent, distorted Dirac cones displaced from high-symmetry points, giving rise to intrinsic self-doping effects.^17,18^ These unique electronic features highlight GYs as promising candidates for next-generation electronic and optoelectronic applications.^22–24^

Motivated by the significant advances achieved through both theoretical predictions and experimental realizations of novel 2D carbon allotropes, considerable effort has been devoted to the search for new members of the GY family with potential for practical applications. In 2020, Mavrinskii and Belenkov proposed new GY structures obtained from 4–6–12 graphene (graphenylene) through the addition of acetylenic groups at specific sites.^25^ Their study, however, was largely limited to structural classification and comparative energetics, leaving open fundamental questions regarding stability and key physical properties such as mechanical, optical, and vibrational. These properties are crucial for validating the feasibility of experimental realization and for guiding potential applications of these structures. Further studies have been initiated to elucidate these properties for select members of this family. Costa Miranda et al. recently confirmed the dynamical stability and semiconducting character of a single member (α – *L*_4–6–12_) of this new family, referred as α-GP-GY in their work.^26^ Other study by Xu et al. explored the CO_2_ capture capability of the structure denoted as β1 – *L*_4–6–12_ in the original classification, reporting high adsorption capacity upon lithium decoration.^27^ Nevertheless, the fundamental physical properties of the latter structure, including its intrinsic stability, mechanical response, and detailed electronic structure, remain largely unaddressed.

In this work, we have conducted a comprehensive characterization of β1 – *L*_4–6–12_. For clarity and to emphasize its distinctive ring topology, we hereafter refer to this structure as Cyclo-graphyne (CGY). This allotrope is composed of sp- and sp^2^-hybridized carbon atoms forming 12-membered rings that alternate between triangular and tetragonal configurations, arranged in a circular motif that gives rise to large 24-membered pores. We systematically investigate its energetic, dynamical, and thermal stability, alongside its electronic, mechanical, optical, and vibrational properties, using first-principles calculations based on density functional theory (DFT).

## Methodology

Electronic structure calculations were carried out using the all-electron Fritz Haber Institute Ab-Initio Molecular Simulations (FHI-AIMS)^28^ code. FHI-AIMS expands the electron density and all the operators over an all-electron numerical-atomic-orbitals basis set. In this work, we have used the “tight” option, which provides safe preconstructed default definitions for the different chemical species. The Perdew–Burke–Ernzerhof (PBE) parametrization of the generalized gradient approximation (GGA)^29^ of the exchange-correlation energy has been adopted throughout. Recognizing that GGA-PBE significantly underestimates the band gap, a portion of the electronic structure calculations was also done using the hybrid Heyd–Scuseria–Ernzerhof (HSE06)^30^ functional. The Broyden-Fletcher-Goldfarb-Shanno (BFGS) algorithm^31^ has been used to relax both the atomic coordinates and the lattice vectors, until the maximum force on each atom was below 10^–3^ eV/Å, and the total energy difference was less than 10^–6^ eV. A Γ-centered Monkhorst–Pack k-point mesh of 32 × 32 × 1 was used for structural optimization and static electronic calculations. A denser 64 × 64 × 1 mesh was employed for the density of states (DOS) computation. A vacuum layer of 20 Å along the out-of-plane direction was used to eliminate spurious interactions between periodic images.

Ab initio molecular dynamics (AIMD) simulations were conducted using the i-PI Python^32^ code as a universal force and trajectory engine, operating in client–server mode. The forces at each time step were computed on-the-fly using the FHI-AIMS code, and then used by i-PI to update the positions of the nuclei. A Nosé–Hoover thermostat^33,34^ was employed to sample the canonical (NVT) ensemble, with a 1 fs integration time step and a total simulation duration of 5 ps.

Phonon dispersion relations were obtained using the finite displacement method as implemented in the Phonopy package,^35^ with input data derived from DFT simulations. From the fully relaxed unit cell, 2 × 2 × 1 supercells incorporating small atomic displacements were generated, and interatomic forces were computed for each configuration. The force constants obtained were then utilized to construct and diagonalize the dynamical matrix, yielding phonon dispersion relations.

To perform optical calculations, the linear macroscopic dielectric tensor ϵ_*ij*_(ω) was computed within the random phase approximation (RPA) framework.^36^ The imaginary part ϵ_2_(ω) of the dielectric function was obtained directly from the interband transitions. The corresponding real part ϵ_1_(ω) was then derived via the Kramers–Kronig transformation.^37^ From these dielectric function components, it is possible to determine key optical properties, such as the absorption coefficient:

$$\alpha \left(\omega \right)=\sqrt{2}\omega {\left[\sqrt{\epsilon_{1}^{2}+\epsilon_{2}^{2}}-\epsilon_{1}\right]}^{1/2}$$ 1

the refractive index:

$$n\left(\omega \right)=\frac{\sqrt{2}}{2}{\left[\sqrt{\epsilon_{1}^{2}+\epsilon_{2}^{2}}+\epsilon_{1}\right]}^{1/2}$$ 2

and the reflectivity:

$$R\left(\omega \right)=|\frac{\sqrt{\epsilon_{1}+i\epsilon_{2}}-1}{\sqrt{\epsilon_{1}+i\epsilon_{2}}+1}{|}^{2}$$ 3

In these equations, ω is the photon energy.

## Results and Discussion

### Structural Properties and Energetic Stability

Figure 1a shows the planar CGY monolayer with a hexagonal unit cell composed of 36 carbon atoms, belonging to the *P*6/*mmm* space group (No. 191). The optimized lattice parameters are *a* = *b* = 13.55 Å, with angles α = β = 90° and γ = 120°. Unlike graphene, which has a primitive hexagonal lattice with uniform C–C bond lengths of 1.422 Å,^38^ the CGY lattice is composed of two distinct types of 12-membered rings that alternate between triangular and tetragonal configurations. Upon periodic repetition of the unit cell, these motifs interconnect in a circular arrangement, giving rise to larger 24-membered pores throughout the lattice. This topology results in five distinct bond lengths ranging from 1.225 to 1.419 Å, and bond angles at the vertices between 112.664° and 129.419°. The lattice parameters and bond lengths are in good agreement with those reported for β1-GY.^25,27^

**Figure 1:**
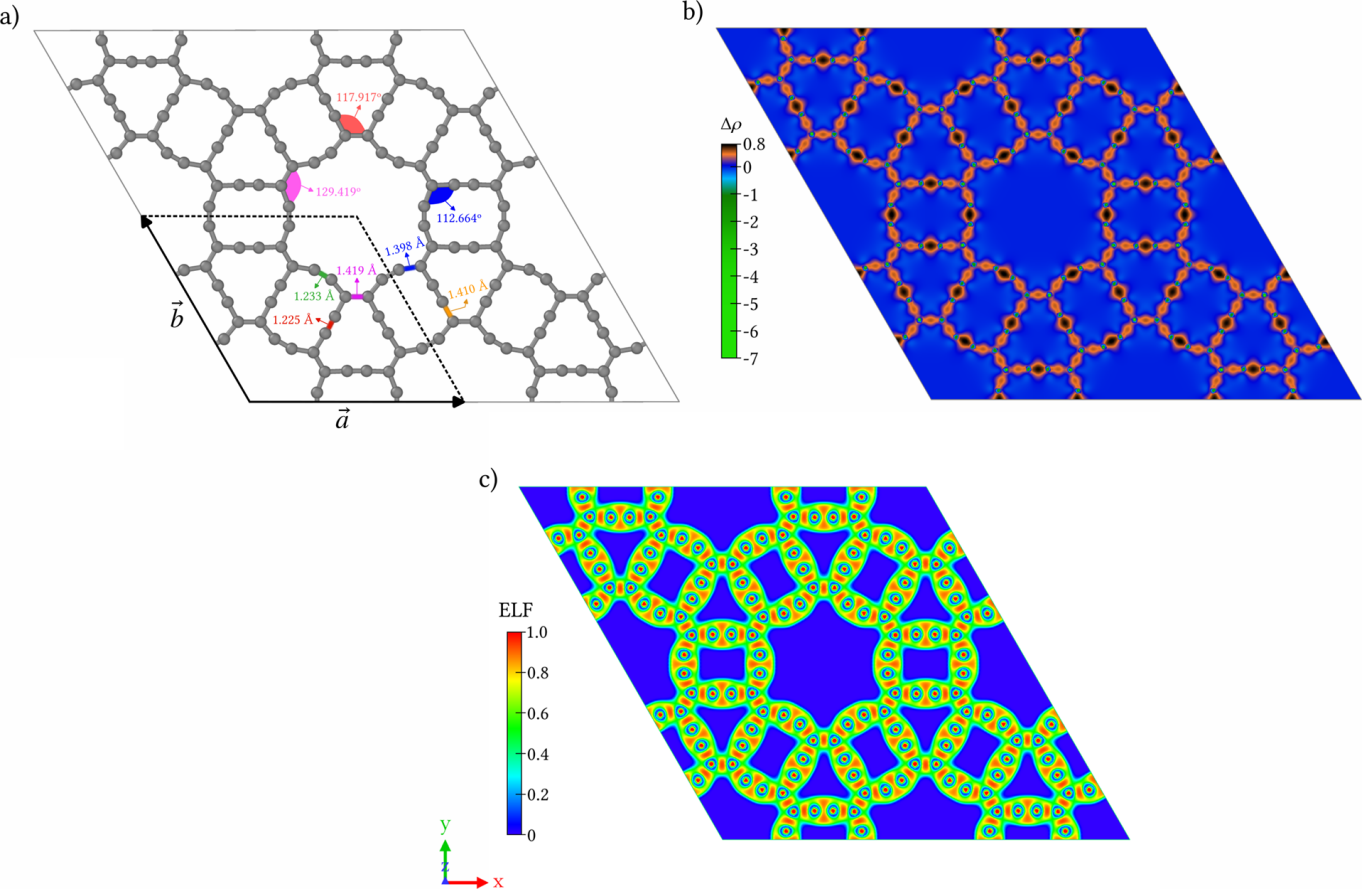
Schematic representation of (a) the CGY monolayer, showing the different C–C bond lengths and bond angles. The hexagonal unit cell and lattice vectors are indicated by the black box. (b) charge density difference, highlighting regions of charge accumulation and depletion, and (c) the electron localization function (ELF), illustrating the nature of chemical bonding across the structure.

The electronic density distribution was further analyzed through the charge density difference (Δρ), defined as the difference between the self-consistent electronic density and the superposition of the densities of isolated carbon atoms. As illustrated in Figure 1b, positive values are concentrated in interatomic regions, indicative of covalent charge accumulation, with particularly strong accumulation along the acetylenic linkages. Negative values, localized around the atomic cores, correspond to charge depletion.

To gain deeper insight into the charge distribution and bonding character of CGY, we analyzed its electron localization function (ELF), shown in Figure 1c. The ELF quantifies electron localization, where values of 1 indicate strongly localized electrons (e.g., in covalent bonds or lone pairs), 0.5 corresponds to a homogeneous electron gas-like distribution, and a value of 0 represents regions with minimal or no electron density. The ELF analysis for CGY reveals strong localization around the sp^2^-hybridized carbon atoms. In contrast, the region in the vicinity of the acetylenic linkages exhibits slightly lower ELF values, indicating greater electron delocalization compared to the sp^2^ bridges. This pattern suggests the coexistence of both sp- and sp^2^-hybridized carbon atoms in the CGY monolayer.

The feasibility of the experimental realization of the CGY nanosheet was evaluated by calculating its formation energy (*E*_form_), defined as

$$E_{\mathrm{form}}=\frac{E_{\mathrm{total}}-N\cdot E_{\mathrm{graphene}}}{N}$$ 4

where *E*_total_ is the total energy of the designed structure, *E*_graphene_ is the energy per atom in graphene, and *N* is the total number of carbon atoms in the structure. To evaluate bonding stability, we calculated the cohesive energy (*E*_coh_) using the expression:

$$E_{\mathrm{coh}}=\frac{E_{\mathrm{total}}-N\cdot E_{C}}{N}$$ 5

where *E*_C_ is the energy of an isolated carbon atom. We have calculated the formation and cohesive energies for other 2D hexagonal carbon allotropes using the same level of theory. In addition, the areal density was computed as the total number of carbon atoms per unit area of the unit cell. The results are presented in Table 1.

**Table 1:** Comparison of Formation Energy (*E*_form_), Cohesive Energy (*E*_coh_), and Areal Density among Representative 2D Carbon Allotropes^a^

structure	*E*_form_ (eV/atom)	*E*_coh_ (eV/atom)	areal density (atom/Å^2^)
graphene	0.00	–9.24	0.38
γ-GY	0.64	–8.60	0.29
γ-GDY	0.76	–8.47	0.24
β-GY	0.84	–8.40	0.23
CGY	0.91	–8.32	0.23
α-GY	0.92	–8.31	0.19
carbyne	0.98	–8.26	

a The values reported in this table were calculated in this study.

From Table 1, we conclude that the formation energy of GY-like structures is considerably higher than that of carbon nanomaterials composed solely of sp^2^-hybridized carbon atoms, such as graphene. This can be attributed to the presence of acetylenic groups, which are less stable than sp^2^ bonds, as evidenced by comparing the formation energies of graphene and carbyne. We further note that the formation energy of CGY is only 0.27 eV/atom and 0.15 eV/atom higher than γ-GY and γ-GDY, respectively, both which have already been experimentally synthesized.^4,9^ This suggests a positive outlook for the experimental realization of CGY, considering recent advances in synthetic routes for carbon nanomaterials. Additionally, the cohesive energy of CGY is comparable to that of other members of the GY family. These results suggest strong interatomic bonding in this material, which is essential for maintaining its structural stability. Finally, the low areal densities observed in GY-like structures indicate high porosity, arising directly from the incorporation of acetylenic groups. Our calculations reveal that CGY possesses a geometric pore size of approximately 8.53 Å. This value is about 23% larger than that of α-GY (6.96 Å) and nearly three times greater than that of graphene (2.85 Å). Notably, this substantial porosity is achieved without compromising structural integrity, as CGY exhibits a cohesive energy essentially identical to that of α-GY. This unique combination of an enlarged pore size and preserved stability positions CGY as a highly promising material for applications in gas capture and separation,^39^ energy storage,^40^ and water purification.^41^

### Dynamical and Thermal Stability

Phonon calculations are a well-established method for assessing the dynamical stability of theoretical new nanomaterials. Figure 2 presents the phonon dispersion of CGY along high-symmetry points of the Brillouin zone. The corresponding phonon-projected density of states (PDOS) for the sp- and sp^2^-hybridized carbon atoms is also presented. The absence of phonon branches with negative (imaginary) frequencies across the entire spectrum confirms the CGY dynamical stability. Furthermore, the presence of high-frequency isolated modes in the range from 60 to 70 THz originates from localized vibrations of the acetylenic linkages, as evidenced by the PDOS. Such modes are commonly observed in other carbon allotropes that contain triple bonds.^42^ Less dispersive modes appear between 25 and 45 THz, with comparable contributions from both carbon hybridizations. This frequency range aligns with that of fully sp^2^-hybridized carbon nanostructures such as graphene^43^ and the biphenylene network.^44^

**Figure 2:**
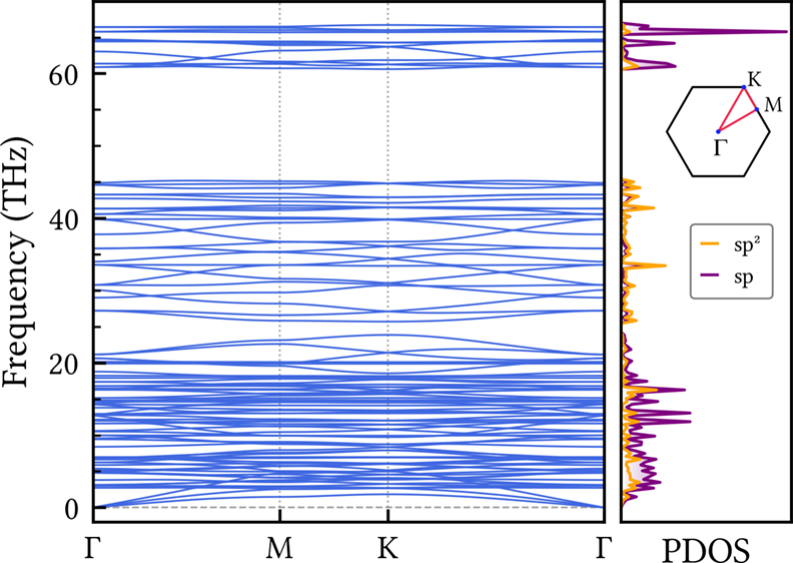
Phonon dispersion and projected density of states (PDOS) of CGY along the high-symmetry directions of the Brillouin zone. The absence of negative frequencies across the entire spectrum indicates the dynamical stability of the structure.

The thermal stability of CGY was evaluated through AIMD simulations at finite initial temperatures of 300 and 1000 K. As illustrated in Figure 3a, the total energy fluctuates around a steady level for both temperatures, indicating the system reaches equilibrium without undergoing structural degradation. In addition, the average temperatures shown in Figure 3b deviate by less than 2% from the target value, reflecting an excellent energy–temperature balance throughout the simulation. These results reveal minimal atomic displacements, no C–C bond rupture, and complete preservation of the planar carbon framework (as shown in the insets of Figure 3a), confirming the thermal stability of the CGY nanosheet up to at least 1000 K.

**Figure 3:**
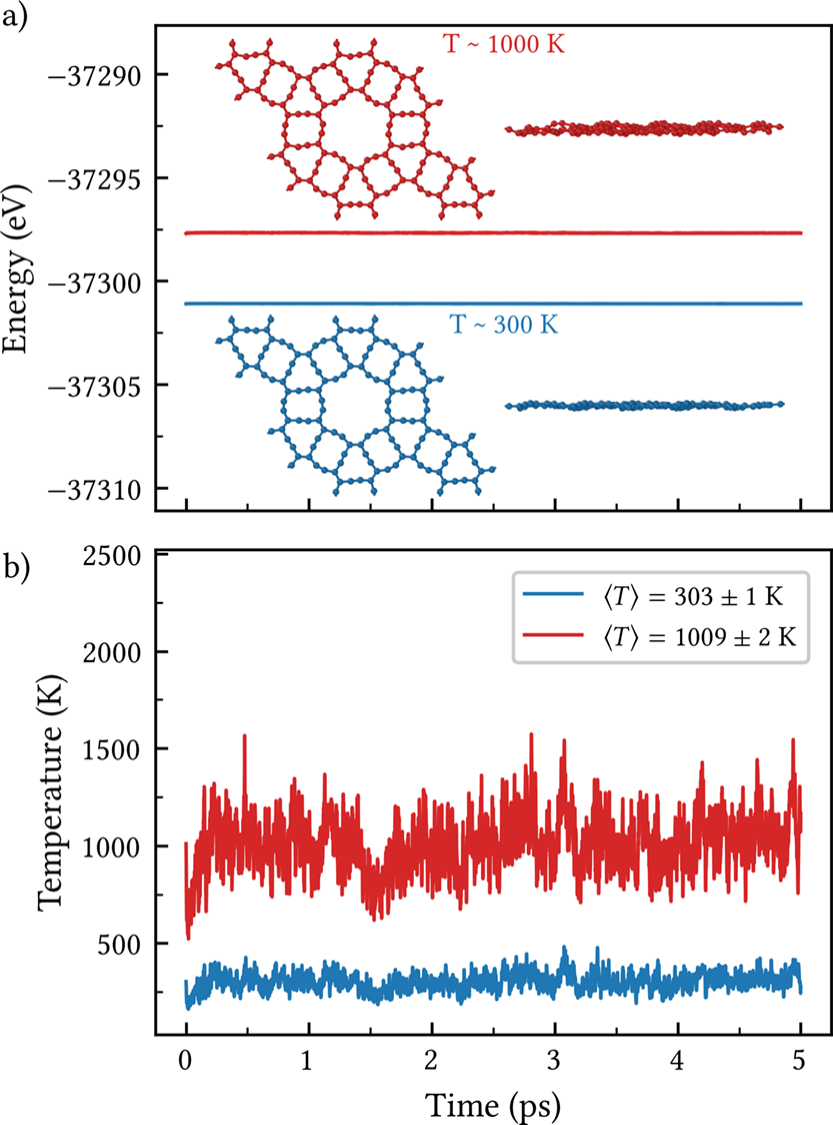
(a) Total energy and (b) temperature fluctuations during AIMD simulations at initial temperatures of 300 K (blue) and 1000 K (red). The insets show the top and side views of the structure at 5 ps.

### Electronic Properties

The electronic band structure of CGY, calculated at the PBE level, is presented in Figure 4a along the high-symmetry points of the Brillouin zone. The corresponding projected density of states (PDOS) for the sp- and sp^2^-hybridized carbon atoms, as well as the total density of states, are also shown. A notable feature is the presence of two Dirac points along the Γ → *M* and *K* → Γ integration paths, indicating that the electrons in CGY behave like massless Dirac Fermions, similar to graphene.^45^ At these two points, the valence and conduction bands touch at the Fermi level, revealing the semimetallic (or zero-gap semiconductor) character of the CGY monolayer. The existence of Dirac cones in GYs has been attributed to the effective hopping induced by acetylenic linkages, which renders GY structures topologically equivalent to graphene.^18^

**Figure 4:**
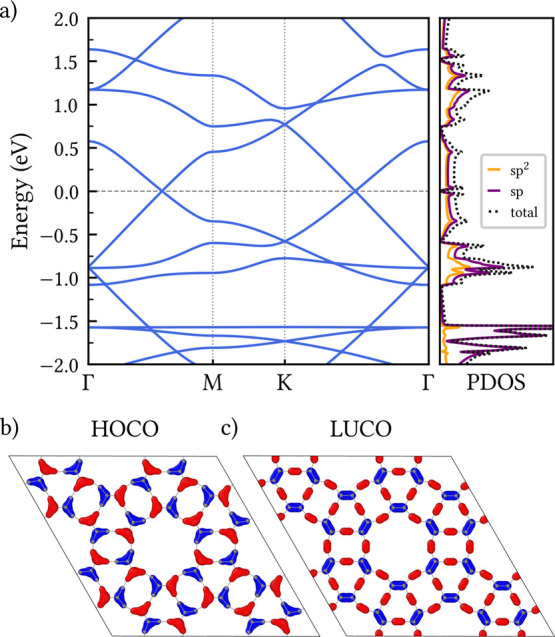
(a) Electronic band structure and projected density of states (PDOS) of the CGY monolayer calculated at the PBE level. The horizontal gray dashed line indicates the Fermi energy. (b, c) Isosurface representations of the highest occupied crystalline orbital (HOCO) and lowest unoccupied crystalline orbital (LUCO), respectively, with red and blue colors denoting different orbital phases.

The PDOS analysis from Figure 4a shows that the contributions from sp- and sp^2^-hybridized carbon atoms are qualitatively similar within the energy window of 1 eV around the Fermi level. However, the sp component is naturally larger than the sp^2^ one in this region, as we have two sp carbon atoms for each sp^2^ site in the CGY structure.

Visual representations of the frontier electronic states are provided in Figure 4, with panel (b) showing the highest occupied crystalline orbital (HOCO) and panel (c) the lowest unoccupied crystalline orbital (LUCO). These states are the main contributors to reactivity and electronic transport in the vicinity of the Fermi level. It is found that the charge density associated with the HOCO and LUCO are spatially complementary. The HOCO exhibits enhanced amplitude on the C^sp^–C^sp^2^^–C^sp^ bridges, whereas the LUCO is strongly localized on the C^sp^–C^sp^ and C^sp^2^^–C^sp^2^^ bonds. This complementary distribution suggests distinct roles of the acetylenic and mixed hybridized bonds in defining the electronic response of CGY.

Considering the known limitations of GGA functionals in accurately predicting band gap values, we further computed the electronic band structure of CGY using the HSE06 hybrid functional, as shown in Figure 5. The HSE06 results exhibit a band structure qualitatively similar to that obtained from the GGA-PBE calculations (Figure 4a). The most noticeable difference is an almost rigid outward shift of the bands relative to the Fermi energy, with the conduction bands moving upward and the valence bands moving downward. However, this shift does not open a significant gap, preserving the two Dirac cones and unambiguously establishing the semimetallic nature of CGY.

**Figure 5:**
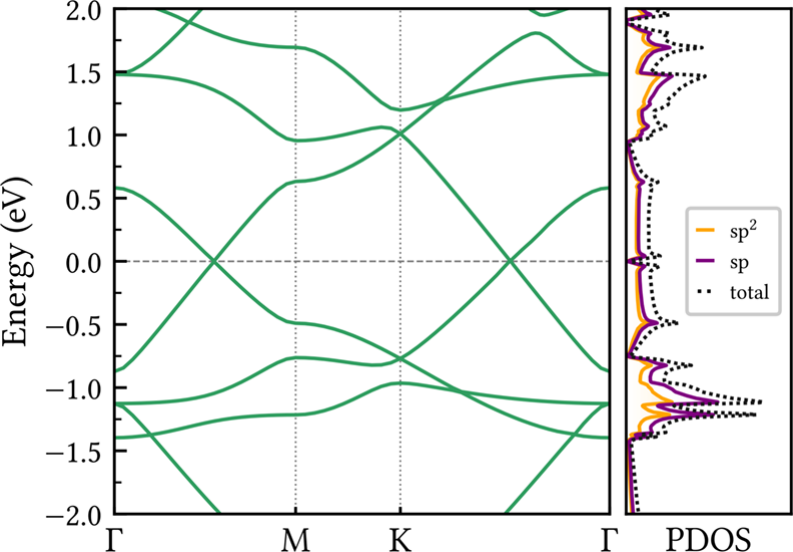
Electronic band structure and projected density of states (PDOS) of the CGY monolayer calculated at the HSE06 level. The horizontal gray dashed line indicates the Fermi energy.

### Mechanical Properties

The mechanical properties of the CGY monolayer were evaluated to assess both its mechanical stability and elastic constants. These calculations were carried out using the energy–strain approach, which analyzes the variation in total energy under small lattice distortions. To this end, in-plane strains were applied by systematically varying the lattice parameters from their unstrained values. We considered strain values in the range −0.01 ≤ ε ≤ 0.01, with increments of 0.005. For these small deformations, the elastic strain energy *U*(ε), defined as the difference between the total energy of the strained and relaxed systems per unit area, is written in terms of strain components according to the following relation:

$$U\left(\varepsilon \right)=\frac{1}{2}C_{11}\varepsilon_{xx}^{2}+\frac{1}{2}C_{22}\varepsilon_{yy}^{2}+C_{12}\varepsilon_{xx}\varepsilon_{yy}+2C_{66}\varepsilon_{xy}^{2}$$ 6

where *C*_11_, *C*_22_, *C*_12_, and *C*_66_ are the components of the stiffness tensor, corresponding to 1 – *xx*, 2 – *yy*, and 6 – *xy* according to the Voigt notation.^46^ This general relation is further simplified for isotropic systems with hexagonal symmetry. Such simplification originates from *C*_11_ = *C*_22_ and the Cauchy relation 2*C*_66_ = *C*_11_ – *C*_12_, yielding:^47^

$$U\left(\varepsilon \right)=\frac{1}{2}C_{11}\left(\varepsilon_{xx}^{2}+\varepsilon_{yy}^{2}+2\varepsilon_{xy}^{2}\right)+C_{12}\left(\varepsilon_{xx}\varepsilon_{yy}-\varepsilon_{xy}^{2}\right)$$ 7

From the computed energy–strain curves, *C*_11_ was extracted via a least-squares fit of the uniaxial deformation data along the *x* direction. Subsequently, *C*_12_ was obtained by combining the fitted *C*_11_ value with the results from equibiaxial strain deformations. Unlike bulk materials, 2D systems lack a well-defined thickness, which is required for the calculation of elastic constants in three-dimensional units. Consequently, the elastic constants of 2D structures are conventionally expressed in N/m, rather than in GPa as in bulk materials.

The stiffness tensor components allow for the calculation of fundamental mechanical properties, including the Young’s modulus (*Y*) and Poisson’s ratio (ν). The former quantifies the resistance of the material to uniaxial tensile stress, within the elastic regime. The latter describes the lateral strain response under uniaxial stress, with lower values indicating less lateral deformation for a given axial strain. Collectively, these parameters reflect the intrinsic bond strength and structural response of the material to mechanical loads. The dependence of these properties on the *C*_*ij*_ components simplifies considerably for isotropic systems,^48^ yielding:

$$Y=\frac{C_{11}^{2}-C_{12}^{2}}{C_{11}},\;\mathrm{and}\;\nu =\frac{C_{12}}{C_{11}}$$ 8

In Table 2 we report the stiffness constants, Young’s modulus, and Poisson’s ratio of CGY and several representative 2D hexagonal carbon allotropes, all calculated at the same level of theory. It is worth noting that these results are in good agreement with those reported in the literature.^13,23,48^

**Table 2:** Stiffness Constants (*C*_*ij*_), Young’s Modulus (*Y*), Poisson’s Ratio (ν), and Areal Density for Selected 2D Carbon Allotropes, All Calculated in This Work

structure	*C*_11_ (N/m)	*C*_12_ (N/m)	*C*_66_ (N/m)	*Y* (N/m)	ν	areal density (atom/Å^2^)
graphene	353.26	62.59	145.33	342.17	0.18	0.38
γ-GY	201.19	84.00	58.60	166.12	0.42	0.29
γ-GDY	153.81	68.60	42.61	123.21	0.45	0.24
β-GY	131.63	88.47	21.58	72.17	0.67	0.23
CGY	74.42	56.94	8.74	30.85	0.77	0.23
α-GY	95.44	83.32	6.06	22.70	0.87	0.19

The Born–Huang stability criterion^49^ states that, for a 2D material to be mechanically stable, the components of the stiffness tensor must satisfy the conditions *C*_11_ *C*_22_ – *C*_12_^2^ > 0 and *C*_66_ > 0. For isotropic hexagonal systems, these requirements simplify to *C*_11_ > |*C*_12_| and *C*_66_ > 0. As shown in Table 2, the calculated stiffness constants of CGY fulfill these conditions, confirming its mechanical stability. Additionally, as with α-GY, the *C*_66_ value for CGY is significantly smaller than its other stiffness tensor components. This arises from the fact that the unit cell area under shear strain varies within a much smaller range than under uniaxial or biaxial deformations.

Further analysis of the data from Table 2 indicates that CGY exhibits intermediate mechanical properties within the GY family, with a Young’s modulus of *Y* = 30.85 N/m and a relatively high Poisson’s ratio of ν = 0.77. Compared to γ-GY/GDY and β–GY, the observed reduction in *Y* and the accompanying increase in ν, reflects the effect of its porous structure and extended acetylenic chains, which allow the lattice to deform more readily under applied stress.^50,51^ As illustrated in Figure 1a, the −C≡C– bridges in CGY are slightly curved, particularly near the tetragonal pores. This geometry suggests that under biaxial strain, deformations are accommodated primarily through bond-angle variations rather than direct bond stretching or compression. Consequently, the *C*_11_ and *C*_12_ components of the stiffness tensor are very close in value, resulting in a high ν.

The trend relating elastic constants to areal density, as observed in Table 2, is further illustrated in Figure 6. The plot of Young’s modulus versus Poisson’s ratio, colored according to areal density, reveals a continuous trend across the GY family, where decreasing areal density is associated with reduced stiffness and enhanced flexibility. The incorporation of acetylenic groups introduces porosity, reduces areal density, and enhances structural flexibility, thereby decreasing the in-plane stiffness, a well-understood mechanism in the literature.^50,51^ Notably, although β-GY share the same areal density, CGY exhibits significantly lower elastic constants. This mechanical softening is attributed to its distinct topology, which incorporates tetragonal and dodecagonal rings absent in β-GY. These findings suggest that the mechanical response of GYs is governed not only by areal density but also by the specific spatial distribution of acetylenic groups across the lattice.

**Figure 6:**
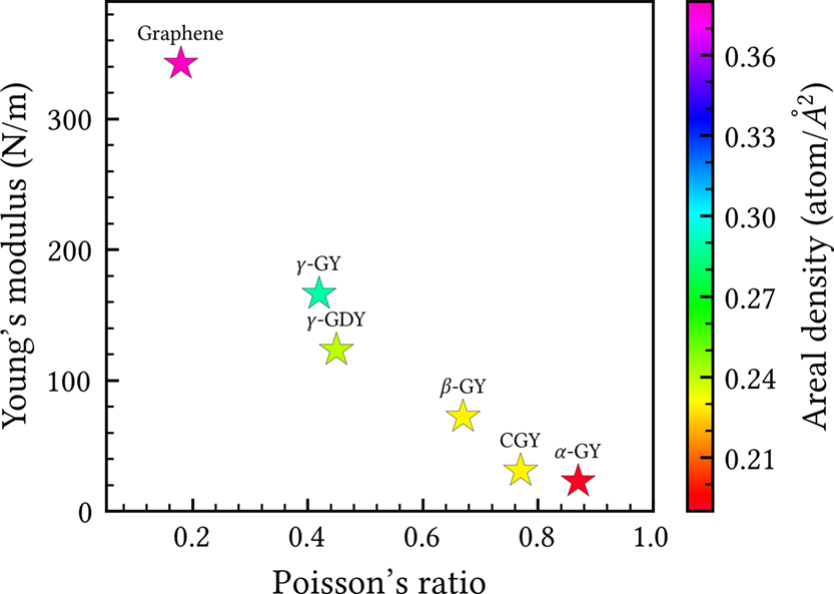
Young’s modulus versus Poisson’s ratio for selected 2D carbon allotropes. The colorbar represents the areal density of each structure.

### Optical Properties

In Figure 7, the optical coefficients of CGY are presented as a function of photon energy. Due to the nearly isotropic nature of the monolayer, the in-plane optical response along the *x* and *y* directions is essentially identical. Unlike other GY allotropes, where absorption spectrum displays finite intensity already near zero photon energy,^14,52^ CGY exhibits negligible absorption below 0.7 eV (Figure 7a). The first prominent feature is a sharp near-infrared (IR) peak, followed by a decreasing trend across the visible spectrum and a subsequent rise to maximum values in the ultraviolet (UV) region. This optical profile suggests that CGY could be particularly suitable for applications in UV-selective photodetectors and optical sensors.

**Figure 7:**
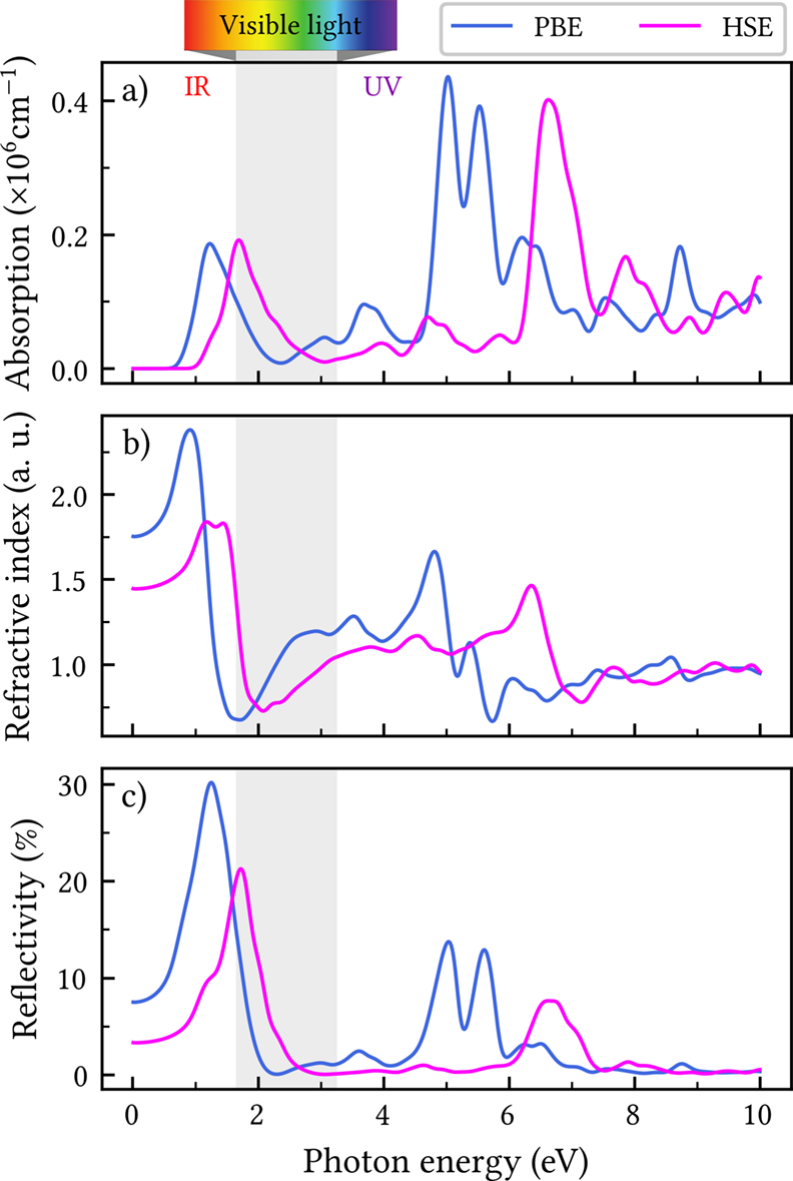
In-plane optical properties of the CGY monolayer: (a) absorption coefficient, (b) refractive index, and (c) reflectivity as a function of photon energy.

The refractive index (Figure 7b) reaches its highest values in the IR region, with lower values in the UV and visible ranges. Similarly, the reflectivity (Figure 7c) is higher in the IR region, reaching approximately 30%. It drops sharply beyond 1.5 eV before increasing again between 4 and 7 eV. The overall reflectivity of CGY is significantly lower than that of β-GY, moderate compared to γ-GY, and similar to α-GY.^14,52^

For all optical coefficients depicted in Figure 7, the HSE06 spectrum is rigidly shifted by approximately 0.5 eV to higher energies relative to PBE. This shift is consistent with the known tendency of hybrid functionals to increase the band gap and partially correct the underestimated transition energies of semilocal functionals.

### Vibrational Properties

Raman and infrared (IR) spectroscopies are powerful experimental techniques for probing bonding characteristics and lattice dynamics in carbon-based materials. Accordingly, the calculated vibrational spectra for CGY, shown in Figure 8, provide a direct fingerprint for future experimental characterization. The Raman spectrum presented in Figure 8a is characterized by four prominent peaks located at 1119, 1337, 2044, and 2095 cm^–1^. The first two low-frequency modes are related to symmetric in-plane vibrations of sp^2^-hybridized carbon atoms. In contrast, the higher-frequency peaks are unambiguous signatures of C≡C stretching vibrations, a characteristic trait of GYs structures.^53^ The atomic displacement pattern for the most intense mode at 2044 cm^–1^, visualized in panel (c), confirms its assignment to symmetric stretching along the triple bonds.

**Figure 8:**
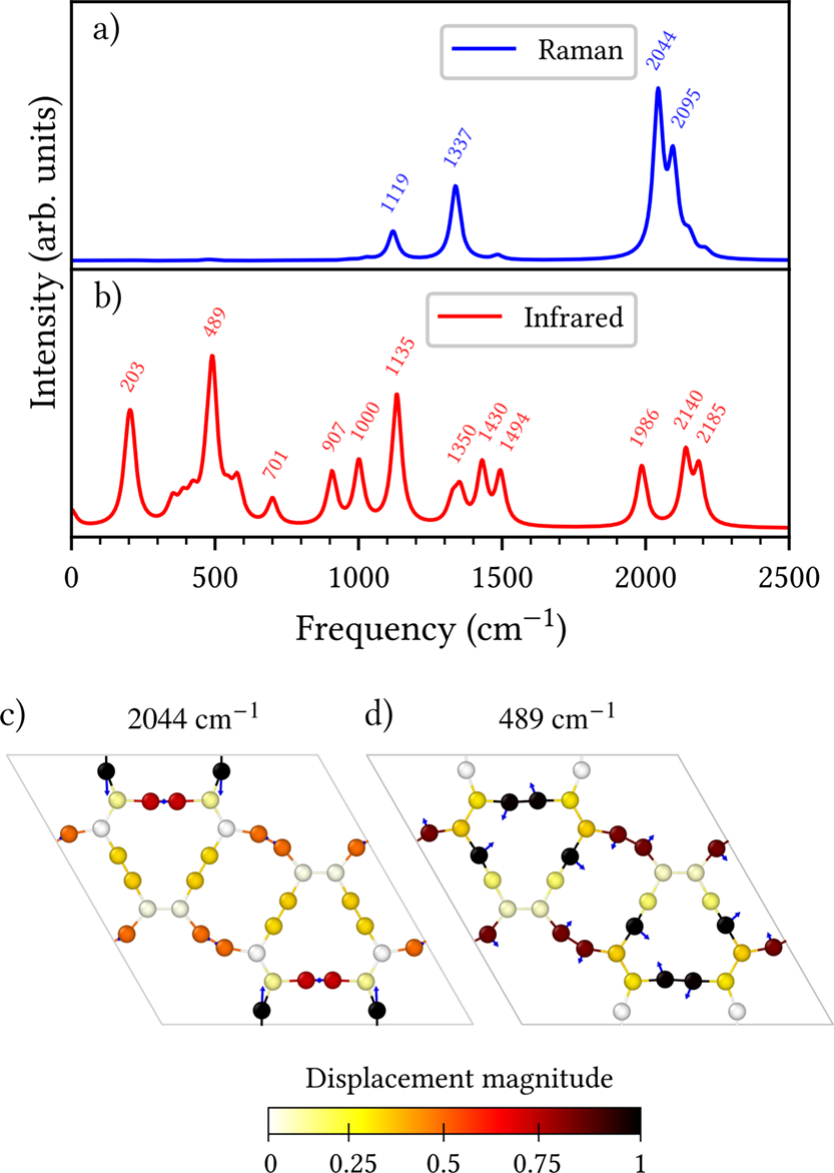
Simulated (a) Raman and (b) infrared spectra of the CGY monolayer with labeled peak frequencies (in cm^–1^). Panels (c) and (d) show the atomic displacement patterns corresponding to the most intense Raman and infrared peaks, respectively. Normalized displacement magnitudes are represented by a white-to-black color scale (inactive to active), and blue arrows indicate the displacement directions.

The IR spectrum shown in Figure 8b exhibits a richer distribution of vibrational modes compared to the Raman spectrum. The lowest-frequency peak at 203 cm^–1^ is attributed to out-of-plane vibrations. The most intense band in the entire spectrum, located at 489 cm^–1^, along with a feature at 701 cm^–1^, arise from symmetric and asymmetric bending vibrations involving the acetylenic linkages. The atomic displacement pattern for this intense 489 cm^–1^ mode is shown in panel (d), confirming its character. Numerous bands between 907 and 1494 cm^–1^ are assigned to collective bending and stretching motions of both sp- and sp^2^-hybridized carbon atoms. Finally, strong IR activity in the high-frequency region at 1986, 2140, and 2185 cm^–1^ are unequivocally assigned to triple bond stretching vibrations.

Taken together, the calculated Raman and IR spectra from Figure 8 provide a comprehensive vibrational fingerprint unique to the CGY monolayer. The distinct high-frequency C≡C stretching modes, particularly the strong Raman peak at 2044 cm^–1^, and the intense IR peak at 489 cm^–1^, serve as unambiguous spectroscopic signatures for identifying this allotrope. These results not only elucidate the lattice dynamics and bonding character of CGY but also establish a crucial reference for its future experimental characterization and differentiation from other GY variants.

## Summary and Conclusions

In this work, we have provided a comprehensive characterization of Cyclo-graphyne (CGY), an emerging two-dimensional carbon allotrope composed of sp- and sp^2^-hybridized carbon atoms. Its structure forms 12-membered rings that alternate between triangular and tetragonal configurations, arranged in a circular motif that gives rise to large 24-membered pores. First-principles all-electron calculations confirm its energetic stability, while phonon dispersion curves and ab initio molecular dynamics simulations demonstrate its dynamical and thermal stability up to at least 1000 K.

Electronic structure calculations reveal that CGY is a semimetal featuring two Dirac cones in its electronic structure. Its hexagonal lattice incorporating acetylenic groups gives rise to nearly isotropic elastic behavior. The high porosity originating from its acetylenic linkages results in a highly compliant structure, with a Young’s modulus approximately 11 times lower and a Poisson’s ratio nearly 4 times higher than those of graphene. Optical simulations reveal a nearly isotropic in-plane response, characterized by strong ultraviolet absorption and pronounced infrared reflectivity. Furthermore, the calculated vibrational spectra provide a unique fingerprint for experimental identification, featuring a few sharp, distinct Raman peaks complemented by a richer array of infrared modes.

In summary, our results highlight CGY as a mechanically flexible yet structurally robust 2D carbon allotrope. Its unique structure, featuring large 24-membered pores, positions CGY as a promising candidate for future applications in areas such as gas capture and separation, flexible nanoelectronics, and optoelectronics.

## Supplementary Material



## Notes

Crystallographic information file (CIF) for Cyclo-graphyne (CIF)
